# Integration of cAMP and TRPV4 Signaling to Optimize Collagen Remodeling for Management of Fibrosis

**DOI:** 10.3390/cells15010056

**Published:** 2025-12-28

**Authors:** Connie Di Raimo, Christopher A. McCulloch

**Affiliations:** 1 Department of Laboratory Medicine and Pathobiology, Faculty of Medicine, University of Toronto, Toronto, ON M5S 3K3, Canada; connie.diraimo@utoronto.ca; 2 Faculty of Dentistry, University of Toronto, Toronto, ON M5G 1X3, Canada

**Keywords:** fibrosis, G protein-coupled receptor, TRP channels, collagen degradation, extracellular matrix, myofibroblast, signal transduction

## Abstract

**Highlights:**

**What are the main findings?**

- Gαs GPCRs generates cAMP, which inhibits fibrotic processes by optimizing intracellular collagen degradation and remodeling.
- TRPV4 activation regulates collagen remodeling under physiological conditions, but when dysregulated, TRPV4 promotes myofibroblast differentiation and reduces β1 integrin expression.

**What are the implications of the main findings?**

- Ca^2+^ influx through TRP channels modulates cAMP levels by regulating phosphodiesterases and adenylyl cyclases, underpinning the interplay between these signaling systems.
- Coordinated activation of the Gαs GPCRs pathway and inhibition of TRPV4 may provide a novel, bimodal approach to control tissue fibrosis.

**Abstract:**

Fibrosis manifests as an excessive accumulation of fibrillar collagen in tissues where secreted collagen exceeds degradation. Myofibroblasts are important contributors to the excessive collagen seen in fibrotic lesions. Accordingly, targeting signaling pathways that enhance collagen degradation and subdue myofibroblast differentiation has the potential to optimize collagen remodeling and improve organ fibrosis. One of the most promising molecular targets for therapeutic development is the G protein-coupled receptor (GPCR) family, which is diverse, cell-type-specific, multi-pass transmembrane receptors that participate in the regulation of extracellular matrix remodeling. GPCRs are categorized into multiple subclasses, some of which activate signaling cascades that can augment or reduce pro-fibrotic processes, depending on which Gα class is activated. Specifically, activation of Gαs GPCR stimulates production of the second messenger, cyclic adenosine monophosphate (cAMP), which generally inhibits pro-fibrotic mediators. A related, second approach for control of fibrosis is the blockade of a specific mechanosensitive, Ca^2+^-permeable channel that is implicated in fibrosis and contributes to myofibroblast differentiation, the transient receptor potential vanilloid type 4 (TRPV4). In health, TRPV4 activation regulates collagen remodeling, but when dysregulated, it promotes pro-fibrotic gene expression through mechanosensitive transcription factors. In this review, we focus on the functions of the Gαs GPCR pathway and TRPV4 activation through the interplay of the second messengers cAMP and Ca^2+^ ions. Ca^2+^ influx modulates cAMP levels by regulating phosphodiesterases and adenylyl cyclases. We consider evidence that Gαs GPCR and TRPV4 signaling pathways interact antagonistically to either promote collagen degradation or to increase the formation of myofibroblasts through signaling that involves cAMP and Ca^2+^ conductance. Coordinated activation of the Gαs GPCR pathway and inhibition of TRPV4 could provide a novel, bimodal approach to control tissue fibrosis.

## 1. Introduction

The mechanical and structural properties of the extracellular matrix (ECM) are precisely regulated by complex signaling pathways that enable matrix homeostasis in healthy tissues. The ECM undergoes continuous and controlled remodeling: dysregulation of this process compromises organ structure and function. Fibrosis manifests as the excessive accumulation of stiffened fibrillar collagen that affects multiple organs, such as the liver, lung, skin, cardiac muscle, kidney, eye, and periodontium [1,2]. Multiple stimuli promote the formation of fibrotic lesions, including tissue exposure to chemicals, radiation, injury, and/or as a side effect of certain drugs. High-prevalence diseases, including cardiac hypertrophy, metabolic dysfunction-associated steatohepatitis, idiopathic pulmonary fibrosis, scleroderma, chronic kidney disease, periodontitis, and tumor invasion, manifest with poorly organized, stiffened arrays of fibrillar collagen [1,3,4,5], which reflects a loss of the balance between matrix synthesis and degradation that is perturbed. The fibrotic lesions of these diseases arise in particular from reduced degradation of fibrillar collagen. Accordingly, understanding the control of intracellular collagen degradation is central to our understanding of fibrotic diseases and in the identification of new targets for the development of anti-fibrotic drugs.

Collagen synthesis and degradation have been extensively characterized in diverse physiological and pathological conditions. In particular, the multi-dimensional aspects of extracellular collagen degradation and uptake continue to be actively explored [6,7]. Collagen is degraded by numerous catabolic pathways that are localized in the extracellular or intracellular space. Extracellular degradation of collagen is directed by matrix metalloproteinases (MMPs) that cleave collagen fibrils into fragments, which are subsequently degraded through intracellular processes [8,9]. Collagen can be degraded intracellularly via three distinct pathways: integrin-mediated phagocytosis, receptor-mediated endocytosis, and macropinocytosis-like uptake. Collagen phagocytosis uses β1 integrin receptors for cell adhesion to fibrils and non-muscle myosin II filaments, and a large cadre of actin-binding proteins to internalize fragmented collagen fibrils [9,10,11]. All three discrete collagen internalization pathways converge on the delivery of collagen to phagolysosomes, vacuolar compartments in which collagen is degraded by lysosomal hydrolases, such as acid-optimal cathepsins. Defining which pathways are involved in enhancing intracellular collagen degradation and in suppressing myofibroblast differentiation are key goals in controlling the pathological accumulation of disorganized collagen that manifests in fibrotic diseases.

G protein-coupled receptors (GPCRs) are implicated in the increase and reduction in fibrosis, depending on which Gα subclass is activated. These proteins are considered promising therapeutic targets for the clinical management of fibrosis. Notably, Gα stimulatory (Gαs) GPCRs stimulate the production of the second messenger cyclic adenosine monophosphate (cAMP), which, in general, slows the progression of fibrosis [3,4,5]. While the potentially anti-fibrotic effect of cAMP could be exploited to increase collagen degradation, we note that ECM remodeling involves multiple, complex processes. These processes must be precisely balanced by multiple signaling systems to maintain matrix homeostasis [1]. Accordingly, therapeutic perturbation of intracellular collagen degradation to reduce fibrosis needs to be carefully managed to avoid unintentional off-target effects, such as excessive ECM degradation.

Anchorage-dependent cells exhibit specialized collagen adhesions (often containing β1 integrins) that act as mechanosensors. These adhesions can “sense” the mechanical properties of the surrounding ECM (e.g., stiffness of collagen fibers). Subsequently, myosin-generated contractile forces enable internalization of individual collagen fibrils. The internalization of collagen fibrils is regulated by multiple processes, including calcium ion (Ca^2+^) conductance through plasma membrane channels [10]. Aberrant function of Ca^2+^-permeable channels and the resultant perturbation of downstream Ca^2+^ signaling pathways are often manifested in fibrotic lesions [12,13,14]. The regulation of Ca^2+^ influx through channels such as transient receptor potential vanilloid type 4 (TRPV4) is important in modulating matrix remodeling. TRPV4 is expressed in multiple cell types and is a polymodal, Ca^2+^-permeable, mechanosensory channel that can detect alterations in the mechanical properties of the ECM. Conversely, TRPV4 affects the mechanical properties of the ECM by its regulation of collagen remodeling [15]. Dysregulation of TRPV4 signaling enhances a pro-fibrotic positive feedback loop in which ECM is pathologically stiffened, which increases matrix stiffness and enhances TRPV4 activation. Ca^2+^ permeation through ion channels like TRPV4 modulates the effects of other second messengers like cAMP. Here, we focus on the functional relationships between the second messengers cAMP and Ca^2+,^ in particular when the balance between matrix synthesis and degradation is perturbed and when ECM structure and function are altered. We discuss the interplay between GPCR and TRPV4 signaling systems in the context of collagen remodeling. We consider how a collective approach to the regulation of cAMP and Ca^2+^ could suggest new treatments for clinical management of fibrotic lesions.

## 2. Physiological Mechanisms of Collagen Remodeling

The ECM comprises dynamic, three-dimensional, non-cellular structures that are present in most types of tissues and undergo tightly regulated remodeling for tissue homeostasis. ECMs are composed of >300 proteins, referred to as the core matrisome, of which fibrillar collagen is the most abundant component [16,17]. Remodeling of fibrillar collagen maintains the mechanical properties, signaling functions, and structural integrity of different tissues [6,9,18]. Collagen synthesis and secretion are exhibited by numerous cell types, including interstitial fibroblasts, macrophages, and tissue-specific cells (e.g., mesangial cells of the kidney) to maintain tissue health. Fibroblasts in connective tissues comprise heterogeneous subpopulations that have been characterized by transcriptomic and proteomic methods and exhibit tissue-specific remodeling traits [19,20]. In healthy tissues, fibroblasts balance synthesis, degradation, and reorganization of fibrillar collagen to preserve tissue structure and function [21]. Several discrete collagen degradation pathways have been identified in the extracellular space and in certain intracellular compartments (Box 1).

Box 1 **Collagen degradation pathways.**

Collagen remodeling is essential for maintaining tissue homeostasis. Collagen can be degraded through extracellular and intracellular pathways. Extracellular degradation of collagen is predominantly performed by matrix metalloproteinases and extracellular cathepsins that cleave collagen fibrils [17]. These collagen fragments can undergo further denaturation in the extracellular space, or the fragments can be internalized by local cells and further degraded by intracellular pathways. The main intracellular pathways for collagen internalization are macropinocytosis, receptor-mediated endocytosis, and phagocytosis, the latter of which is mediated by β1 integrins. Macropinocytosis and receptor-mediated endocytosis primarily internalize cleaved soluble collagen fragments, while β1 integrin-containing adhesion receptors serve as the initial sites where phagocytosis leads to the internalization and eventual degradation of intact collagen fibrils [6]. All three intracellular collagen degradation pathways converge on lysosomes, organelles in which collagen is degraded by acid-optimized cysteine proteases, including cathepsins [6,18].

## 3. Pathways of Collagen Degradation

### 3.1. Extracellular Collagen Degradation

The extracellular collagen degradation pathway is dominated by the catalytic activity of matrix MMPs and, in some cell types (such as fibroblasts), by the lysosomal cysteine protease, cathepsin K [22,23,24]. MMPs are a family of zinc endopeptidases that exhibit collagenolytic activity and have been extensively characterized in the context of ECM degradation in various disease states [25]. Fibrillar collagen is resistant to most proteolytic enzymes and can be cleaved only by a subset of MMPs that include enzymes with triple helicase activity, such as MMP-1, MMP-8, MMP-13, and MMP-14 [8,25]. The extracellular MMP pathway generates collagen fragments that can be further degraded by gelatinases such as MMP-2 or MMP-9; collagen fragments may also be degraded intracellularly. In this review, we focus on the control of intracellular collagen degradation since the extracellular pathway has been examined in considerable depth (see reviews [25,26]).

### 3.2. Intracellular Pathways of Collagen Degradation

**Figure 1:**
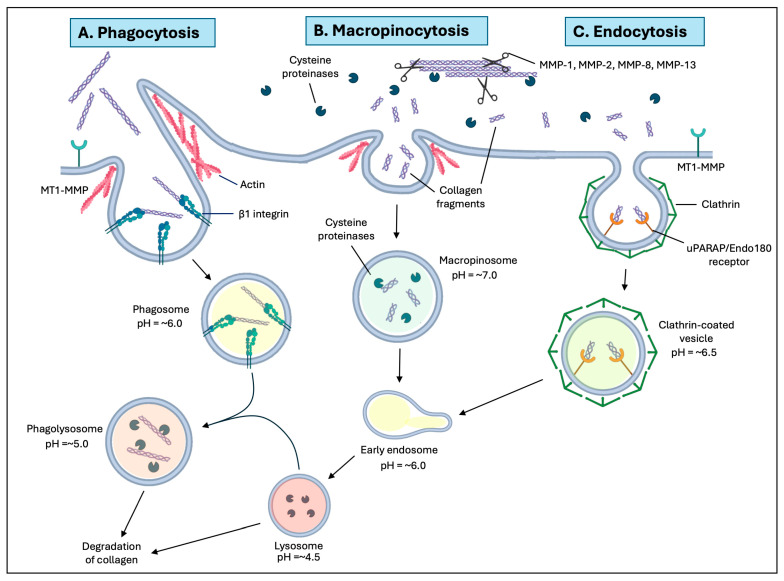
Collagen Degradation Pathways. Collagen can be degraded by extracellular and intracellular pathways. Extracellular collagen digestion is performed by a collection of matrix metalloproteinases (MMPs) and extracellular cathepsins. MMPs and extracellular cathepsins cleave collagen fibrils into collagen fragments, which can be internalized and degraded by intracellular pathways. The main intracellular pathways are phagocytosis, macropinocytosis, and receptor-mediated endocytosis. (**A**) Intact collagen fibrils are internalized by collagen phagocytosis. Initial cleavage of pericellular collagen is performed by membrane-type 1 MMP (MT1-MMP). β1 integrins mediate recognition and fibril adhesion. Actin remodeling enables the formation of the phagocytic cup to internalize fragments into a phagosome. Phagosomes (pH = 6.0) fuse with an acidic lysosome (pH = 4.5) to form a phagolysosome, where collagen fibrils are ultimately degraded. (**B**) Cleaved collagen fragments can be internalized by actin-mediated macropinocytosis. Once internalized, collagen fragments are enclosed in a macropinosome (pH = 7.0), which is then trafficked to early endosomes and then to lysosomes for degradation. (**C**) Collagen fragments can also be degraded through receptor-mediated endocytosis. uPARAP/Endo180 receptors recognize collagen fragments and internalize collagen using clathrin-coated pits to form vesicles (pH = 6.5), which are subsequently trafficked to the lysosome for degradation. All three of the intracellular collagen degradation pathways converge on lysosomes. In these organelles, collagen is degraded by acid-optimized cysteine proteases, including cathepsins.

The intracellular pathways of collagen degradation are distinct and can process previously cleaved and intact fibrillar collagen located in the extracellular space. Pre-cleaved collagen fragments can be internalized by macropinocytosis, or for soluble collagen, by receptor-mediated endocytosis (Figure 1). The initial steps of phagocytosis of intact collagen fibrils are largely reliant on β1 integrin-mediated collagen binding; the internalized collagen is subsequently degraded by lysosomal cysteine proteases such as cathepsins (Figure 1) [9]. β1 integrin activation also promotes the expression of the dipeptidase prolidase through the mitogen-activated protein kinase (MAPK) pathway [27]. Prolidase catalyzes the hydrolysis of dipeptides that contain proline or hydroxyproline on the C-terminal [28,29]. Prolidase recycles collagen and is regulated by surface receptor–ECM interactions, as its activity increases with cell growth and density [28,29]. Additionally, nascent collagen can also be degraded by autophagocytosis, in which newly synthesized molecules are destined for destruction prior to secretion and are enclosed in double membrane-bound autophagosomes. These collagen fragments are further degraded by cysteine proteases after the fusion of autophagosomes with lysosomes. Macropinocytosis is an actin-dependent, endocytic uptake pathway by which soluble collagen enters into large vesicles (macropinosomes) [9]. Macropinocytosis is often activated in response to stimulation by growth factors and is also seen in cells with dysregulated signaling (e.g., cancer) and in cells and tissues in which there is rapid ECM remodeling [30,31,32].

In a separate and distinct pathway, pre-cleaved collagen can be internalized via the receptor-mediated endocytic pathway, which involves the urokinase plasminogen activator receptor-associated protein (uPARAP/Endo180) and the formation of clathrin-coated vesicles [33]. uPARAP/Endo180 is a type-1 membrane protein of the mannose receptor family that binds directly to collagen and is expressed in mesenchymal cells, including subpopulations of fibroblasts, osteoblasts, and chondrocytes [33,34,35]. Following internalization of collagen fragments, clathrin-coated vesicles transit to early endosomes and subsequently mature into terminal lysosomes, where collagen is degraded by cysteine proteases [36,37]. Extracellular collagenases and the uPARAP/Endo180 receptor cooperate to efficiently endocytose collagen in fibroblasts [38]. As uPARAP/Endo180-mediated endocytosis occurs independently of the phagocytosis of largely intact fibrils, these pathways are considered to be fundamentally distinct [39,40].

## 4. Collagen Phagocytosis

### 4.1. Collagen Recognition, Focal Adhesion Formation, and Actin Network Formation

**Figure 2:**
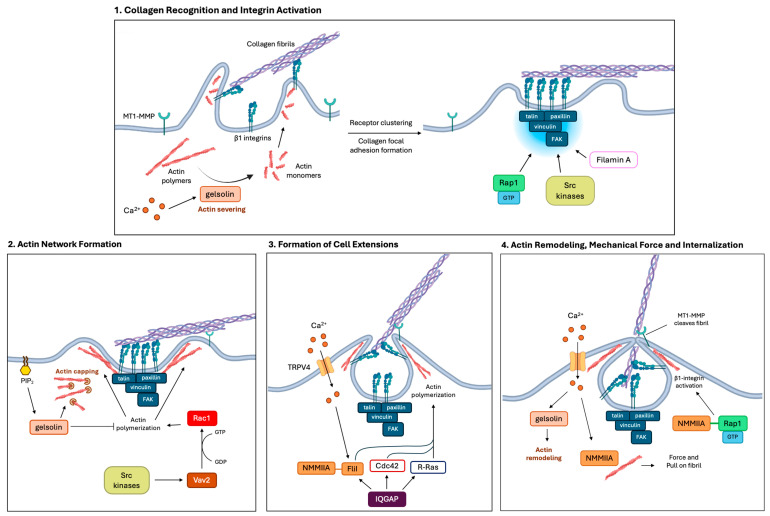
Multistep, regulated process of collagen phagocytosis. Collagen phagocytosis requires recognition, integrin activation, actin remodeling, formation of cell extensions, and mechanical force to allow for internalization. (1) Collagen recognition and integrin activation. Initial cleavage of collagen is mediated by MT1-MMP. The cell binding to collagen fibrils involves β1 integrin recognition as well as Ca^2+^-dependent actin severing in focal adhesions by gelsolin. Integrin binding to collagen fibrils promotes receptor clustering and the activation of Rap1, Src, and talin. This leads to the formation of focal adhesion complexes, organelles in which the signaling proteins FAK, Src, and filamin A promote β1 integrin activation. (2) Actin network formation. At early stages of actin network assembly in the formation of the phagocytic envelopment of fibrils, Src family kinases phosphorylate Vav2, which activates Rac1. GTP-bound Rac1 promotes actin polymerization and the further development of the phagocytic cup, which surrounds the fibril. As the phagocytic cup forms, PIP_2_ accumulates, which enables gelsolin to uncap actin filaments and promote actin assembly. (3) Formation of cell extensions. Actin-rich cell extensions are generated by the activities of IQGAP, which promotes actin polymerization through FliI, Cdc42, and R-Ras. TRPV4 Ca^2+^ influx regulates the interaction between NMMIIA and FliI to generate cell extensions and enhance fibril internalization through actomyosin-dependent membrane remodeling. (4) Actin remodeling, mechanical force, and internalization. Localized TRPV4 Ca^2+^ bursts activate gelsolin’s actin-severing function. Gelsolin also binds to full-length NMMIIA at collagen adhesions. NMMIIA assembles into filaments that bind to the actin filament network and increase localized contractile force. These activities are also synergized through the interaction of Rap1 with NMMIIA for additional β1 integrin activation. MT1-MMP cleaves the pericellular collagen, which promotes the entry of the cleaved fibril into the phagosome.

As described above, intact collagen fibrils are internalized by collagen phagocytosis, which is mediated by β1 integrin-containing collagen receptors [41,42]. The initial cleavage of pericellular collagen fibrils is mediated by membrane-type 1 MMP (MT1-MMP), which is a rate-limiting enzyme for collagen phagocytosis [43]. Fibroblasts recognize collagen fibrils by binding to GFOGER sequences using the cell-surface, heterodimeric transmembrane α2β1 or α11β1 integrins [44,45,46]. Collagens that are not assembled into fibrils preferentially bind to the α1β1 and α10β1 integrins [44,45,46,47]. Integrin binding to collagen fibrils promotes receptor clustering and the activation of Rap1, Src, and talin, which in cultured cells lead to the formation of focal adhesion complexes [48,49]. Cell binding to collagen fibrils also involves Ca^2+^-dependent actin filament severing in focal adhesions by the actin-binding protein, gelsolin (Figure 2) [50]. Collectively, the recognition of collagen and the assembly of signaling-competent adhesion complexes are important rate-limiting steps for phagocytosis of collagen fibrils [42].

Integrin-based focal adhesion complexes contain a large cadre of scaffolding proteins and adaptors that bind actin filaments (i.e., talin, vinculin, paxillin, filamin, α-actinin, zyxin) and signaling proteins such as the focal adhesion kinase (FAK), integrin-linked kinase, and Src [51,52]. Through its interaction with filamin A, talin contributes to β1 integrin activation. The integrin activation step enables high-affinity binding to collagen fibrils [53], which is an important determinant of the initiation of both collagen phagocytosis and synthesis. Focal adhesions are mechanosensing signaling hubs that control how integrins bind to the ECM and integrate with the actin cytoskeleton [10]. The maturation, protein composition, and the regulatory mechanical cues that determine focal adhesion complex function, modify whether fibroblasts favor collagen synthesis or phagocytosis [54]. When ECM polymers are subjected to tensile forces, the formation of focal adhesion complexes is transient and promotes ECM engulfment [10,54]. Meanwhile, mechanosensitive transcription factors remain sequestered in the cytosol [10,54]. Additional regulation of collagen phagocytosis is associated with collagen-bound proteoglycans (e.g., decorin) and glycoproteins (e.g., fibronectin), which can modulate collagen interactions with its cognate receptors. Collagen may also interact with other, non-collagen-binding integrins to promote phagocytosis [55]. Notably, the collagen-binding capacity of β1 integrin-containing adhesions is affected by non-collagenous proteins that coat the surface of the fibrils rather than the core of the collagen molecules [55].

Following collagen recognition and integrin activation, fibroblasts exhibit actomyosin remodeling to enable internalization of collagen fibrils [56,57]. This process involves the small GTPase Rac1 and a constellation of actin-binding proteins (e.g., gelsolin) that contribute to remodeling of sub-cortical actin filaments and engagement of the fibril internalization process [56,57]. The switch from RhoA to Rac1 favors collagen phagocytosis instead of collagen synthesis [56,57]. At early stages of actin network assembly, which is manifest in the phagocytic envelopment of collagen fibrils, Src family kinases phosphorylate the guanine nucleotide exchange factor (GEF) Vav2 [56]. This step is followed by Vav2-mediated activation of the small GTPase Rac1 to promote actin polymerization and the further development of a phagocytic cup to surround the collagen fibril [56]. As the collagen fibril phagocytic cup forms, phosphatidylinositol 4,5-bisphosphate (PIP_2_) accumulates at the site of internalization, which enables gelsolin to uncap the barbed end of actin filaments and mediate rapid actin assembly at the cup [50,58].

### 4.2. Mechanism of Collagen Internalization

For efficient internalization of collagen, actin-rich cell extensions are generated through the activities of the small GTPase IQGAP1, which interacts with Cdc42, R-ras, and flightless-1 (FliI). These signals promote the formation of extensions that surround the fibril, which is followed by internalization [59,60]. Mechanical force is also required to physically pull the fibril inside the cell [10,50,61,62]. This process relies on Ca^2+^-dependent, actomyosin contractility [10,50,61,62]. The mechanical tension generated by contractile forces applied through integrins to collagen fibrils activates mechanosensitive TRPV4 channels that allow localized influx of extracellular Ca^2+^ at the phagocytic site [61]. Here, Ca^2+^ influx through TRPV4 channels regulates the interaction between non-muscle myosin IIA (NMMIIA) and Flil to generate cell extensions that are important for fibril envelopment [61]. Localized Ca^2+^ bursts activate gelsolin’s actin-severing function, which enhances collagen binding to previously unoccupied β1 integrin-containing adhesions [57]. Gelsolin also binds to full-length NMMIIA at collagen adhesions in a Ca^2+^-dependent manner [10,11]. NMMIIA assembles into filaments that bind to the actin filament network and mediate the generation of localized contractile forces. These activities synergize through the interaction of Rap1 with NMMIIA, which promotes additional β1 integrin activation [10,48]. Collectively, these processes promote cell adhesion to collagen, activate Ca^2+^ entry through plasma membrane channels (e.g., TRPV4), and enhance fibril internalization through actomyosin-dependent membrane remodeling (Figure 2) [10,11,48].

Fibroblasts initially engulf part of the fibril using actin-rich pseudopods at matrix adhesion sites [35]. Once internalized, the membrane-enclosed fibril (in a phagosome) fuses with a lysosome to create a phagolysosome, a vacuolar compartment in which collagen fibrils are degraded by lysosomal enzymes that include cysteine proteases [63,64,65,66]. Certain lysosomal cysteine proteases exhibit efficient and robust collagenolytic activity, including cathepsins L, K, S, and B [23,65,67,68,69]. In theory, deficiencies in the activation, catalytic function, localization, and local pH associated with any cathepsins that degrade collagen will reduce the effectiveness of collagen digestion and contribute to fibrosis [24,70,71,72].

## 5. Dysregulation of ECM in Fibrotic Lesions

Small perturbations of the signaling modules that control collagen remodeling often contribute to long-standing, chronic disease and to alterations in organ structure and function. Healthy ECM remodeling requires a careful balance between collagen synthesis and degradation, which is dysregulated in fibrotic diseases, connective tissue disorders, muscular dystrophy, and invasive cancers (Box 2) [73,74]. Fibrosis is a major pathological feature of diseases that contributes to ~35% of global deaths (2019) [75].

Box 2 **Chronic wound healing leads to fibrosis.**

Fibrosis manifests as an excessive and progressive accumulation of ECM components, such as collagen and fibronectin. These imbalances are frequently found in inflamed or injured tissues [71]. The production of excessive ECM disrupts normal tissue and organ architecture, leading to permanent scarring and/or organ dysfunction [12,71,72]. Aberrant ECM deposition can result ultimately in death, as seen in end-stage liver disease, kidney disease, idiopathic pulmonary fibrosis, and heart failure [72]. Fibrosis affects multiple organ systems, such as skin, lungs, heart, kidneys, pancreas, and periodontal tissues [7,73].

Fibrosis is often associated with chronic inflammation and frequently manifests as excessive accumulation of ECM. The drivers for fibrotic lesions include a wide array of stimuli, including persistent infections, allergic reactions, autoimmune responses, tissue exposure to chemicals, radiation, injury, or side effects of certain drugs (Table 1) [76]. There has been a rise in cases of drug-induced pulmonary fibrosis, which has become increasingly prevalent in respiratory disability [77,78]. Drugs that lead to fibrosis provide a useful method to understand the complex underlying mechanisms of fibrosis. Tissues that sustain repeated injury and/or exhibit chronic inflammation are more likely to manifest fibrosis, partly due to continuous and overactive wound healing processes [79]. For example, in drug-induced periodontal diseases, which are high-prevalence disorders in adults, fibrosis manifests as the formation of dense, stiffened, dysfunctional tissues that fail to effectively seal around the necks of teeth [80].

## 6. Drivers and Cellular Mediators of Fibrosis

An important, pleiotropic cytokine in wound healing that also promotes the formation of fibrotic lesions is transforming growth factor-β1 (TGF-β1) [1,204,205,206,207]. TGF-β acts through canonical SMAD signaling and non-canonical mitogen-activated protein kinase (MAPK)/JNK/p38 signaling pathways to regulate the expression of pro-fibrotic genes [208]. TGF-β1 potently stimulates the accumulation of the ECM, regulates inflammation, and can enhance α-SMA expression in myofibroblasts; all of these factors promote fibrosis [209,210]. Mechanosensors such as stretch-activated membrane channels (e.g., TRPV4 and Piezo1) can be mechanically activated in myofibroblasts and thereby increase cytosolic Ca^2+^ [211,212]. In addition to the effect of increased cytosolic Ca^+2^ on collagen phagocytosis (see above), increased intracellular Ca^2+^ activates Rho-associated protein kinase (ROCK) signaling, which results in enhanced actomyosin contractility [211,212]. Notably, environmental stimuli such as repeated cyclic strain, shear stress, and enhanced ECM stiffness can activate TRPV4, which in turn, enhances ECM production [208,213]. Further, increased Ca^2+^ influx through TRPV4 has been spatially associated in cells with locally high abundance of the fibrillar collagen-binding discoidin domain receptor 1 (DDR1) [214]. DDR1 increases collagen compaction and alignment driven by its association with Ca^2+^-dependent NMMIIA, which drives localized collagen stiffening at sites of cell attachment [214]. As pathological ECM remodeling is a key characteristic of fibrosis, myofibroblasts are therefore of central importance in the excessive production of stiffened collagen fibrils in these lesions.

## 7. Role of the Myofibroblast in Fibrotic Lesions

In many fibrotic diseases, a key culprit in fibrotic ECM generation and remodeling is the persistent activation and recruitment of myofibroblasts [215]. These cells exhibit certain prominent features of the mesenchymal lineage, including expression of vimentin intermediate filaments and the synthesis and secretion of collagens I and III at high levels [216]. During normal tissue repair, locally generated signals can promote the conversion of fibroblasts to myofibroblasts, which exhibit specialized ECM contractile machinery that accelerates wound closure of damaged tissues [2]. Indeed, one of the critical phenotypic features of myofibroblasts is their ability to reorganize and condense the ECM, which alters the mechanical properties of tissues (i.e., increased stiffening) during tissue repair.

The phenotypic transition of relatively non-contractile fibroblasts into highly contractile myofibroblasts is temporally associated with increased transcription of muscle-associated genes like *ACTA2*, which codes for the contraction-associated actin isoform, α-smooth muscle actin (α-SMA) [215]. In fibrotic lesions, excessive synthesis of disorganized collagen fibrils along with the formation of abundant, highly aligned and cross-linked fibrils, fundamentally alters the mechanical properties and function of local tissues [2,217], which is further aggravated by reduced collagen degradation [74]. The formation of a stiff, fibrotic matrix promotes myofibroblast activation, which can generate a positive feedback loop that synergistically increases the deposition of a fibrotic ECM and pathological remodeling [2,215,217,218,219].

### Signals That Activate Myofibroblast Formation

Multiple pathways regulate fibroblast-to-myofibroblast conversion. In this context, we note that many downstream mechanosignaling events converge, leading to the activation of the pro-fibrotic transcription factors myocardin-related transcription factor A (MRTF-A), Yes-associated protein 1 (YAP), and transcriptional coactivator with PDZ-binding motif (TAZ) [2,208,211,216]. YAP, TAZ, and MRTF-A translocate to the nucleus in cells that are subjected to increased mechanical cell stress. In the nucleus, YAP and TAZ act as transcriptional co-activators that drive the expression of pro-fibrotic genes like *COL1A1*, *COL1A2*, *CTGF,* and *ACTA2,* and the upregulation of each other [220,221,222,223]. Following nuclear localization, YAP/TAZ regulates gene transcription through its association with DNA-binding TEA domain family members (TEAD 1–4) [224,225,226]. In unstressed cells, MRTF-A is usually bound to actin monomers and remains in cytosolic sites. However, when the level of actin monomers is reduced because of actin assembly into filaments, MRTF-A enters the nucleus to enhance pro-fibrotic gene transcription, which includes increased expression of α-SMA [211,227]. In contrast with MRTF, YAP/TAZ is mainly regulated through the Hippo pathway (Box 3) by several proteins, including the large tumor suppressor kinase 1 (LATS1/2).

Box 3 **The Hippo pathway regulates YAP/TAZ localization.**

The Hippo pathway is a serine/threonine kinase cascade consisting of MST1/2, SAV1, LATS1/2, YAP, and TAZ [89]. When the Hippo pathway is active, YAP/TAZ is sequestered to the cytosol by phosphorylation of LATS1/2 [82]. When YAP/TAZ is localized to the cytosol, it is inactive as a result of its interaction with scaffold protein 14-3-3. It is subsequently degraded in proteosomes [90]. Discrete mechanical and proliferative cues inhibit LATS1/2-mediated phosphorylation of YAP/TAZ, which then enables nuclear localization [91]. YAP/TAZ associates with the TEAD binding domain to promote cell proliferation, stem cell self-renewal, and the expression of pro-fibrotic genes [89].

In fibrotic lesions, YAP/TAZ promotes matrix synthesis, focal adhesion formation, stress fiber assembly, and sustained expression of β1 integrins in collagen adhesions by activated myofibroblasts [2,5,54,223,228,229]. Upstream of MRTF-A and YAP/TAZ, GPCRs regulate the activity of these transcription factors, which depends on the particular type of heterotrimeric G protein [223,230,231,232] that is involved in the specific signaling system. Certain GPCRs and members of the transient receptor potential (TRP) channel family exhibit mutually interactive regulatory relationships [233,234,235]. For example, functional reciprocity is exhibited by TRPV4, which is affected by the Gαq/11-coupled GPCR angiotensin II receptor type 1 (AT1R) and vice versa [236]. In this instance, AT1R modulates TRPV4 by promoting β-arrestin-dependent inhibition and internalization of TRPV4 (which dampens its activity), while TRPV4 inhibits G protein-associated diacylglycerol and inositol-1,4,5-triphosphate accumulation [236]. When GPCRs and TRP channels are co-expressed in the same cells, the expressed channels can affect GPCR desensitization [236]. Collectively, certain GPCRs, and TRPV4 in particular, may regulate one another and thereby play multiple, complex roles in fibrosis by activating YAP/TAZ and MRTF-A.

## 8. Why Study GPCR and TRPV4 Signaling in the Context of Fibrosis?

In fibrotic lesions, imbalances of collagen remodeling favor the net accumulation of collagen in which synthesis exceeds degradation. Therapeutic interventions that can specifically target collagen degradation **and,** in a complementary fashion, reduce myofibroblast differentiation have not yet been established. This shortcoming is in part due to the fact that the potential interactions and overlap between their respective signaling pathways are not well-defined. GPCRs are a diverse set of receptors and have been one of the most successfully targeted molecular families for the purpose of drug development [237] (e.g., losartan, an angiotensin receptor blocker [238]; ONO-0260164 a prostaglandin EP4-receptor agonist [239]; and fenoldopam, a selective dopamine D1-receptor agonist [240]). They activate a broad array of signaling pathways, which are dependent on the Gα subclass that is coupled with a specific GPCR of interest. Multiple Gα subclasses have been implicated in reducing fibrosis, which underpins their potential for the development of new therapies for clinical management of fibrotic lesions [223].

Gαs GPCRs generate a key anti-fibrotic second messenger, cAMP [5,241]. Downstream signaling initiated by cAMP suppresses collagen production and fibroblast proliferation, and increases intracellular collagen degradation [3,242,243]. Conversely, and as described above, a prominent mechanosensor, TRPV4, is a pro-fibrotic mediator that regulates myofibroblast differentiation, ECM synthesis, cellular contractility, MMP activity, and cytokine production [244]. Evidently, TRPV4 channels can contribute to the progression of fibrosis through their ability to affect mechanotransduction, TGF-β1 stimulation, and Ca^2+^ signaling [244].

In general, GPCRs have a mutual regulatory relationship with TRP channels. But their interactions that affect downstream signaling pathways are not well-defined [236]. In this review, we consider downstream signaling from Gαs GPCRs and TRPV4 in the context of collagen remodeling and their respective second messengers, cAMP and Ca^2+^. The second messenger cAMP is involved in regulating physiological collagen remodeling, in part by reducing lysosomal pH and enhancing the collagenase activity of cathepsins. In contrast, Ca^2+^ is involved in mediating the interaction between gelsolin and NMMIIA [11,242], a contractile protein that is important for the initial steps in collagen internalization. Ca^2+^ also regulates Ca^2+^/calmodulin-dependent phosphodiesterases (PDE) and Ca^2+^-sensitive adenylyl cyclases (AC) [245]. We posit that Ca^2+^ influx through TRPV4 regulates Ca^2+^-sensitive PDEs and adenyl cyclases that directly affect cAMP levels. Thus, in fibrotic lesions, excessive Ca^2+^ influx through TRPV4 would activate PDEs to reduce cAMP levels [246,247,248,249].

## 9. GPCRs Are Therapeutic Targets in Fibrosis

GPCRs are the largest class of cell surface receptors that transduce extracellular cues into intracellular signals as part of cellular responses to diverse stimuli [250,251]. Upon activation, GPCRs undergo allosteric modifications that enable them to interact with one or more of the four major and unique trimeric G protein families: Gαi/o, Gαq/11, Gα12/13, and Gαs [252,253]. In healthy connective tissues, GPCR signaling in fibroblasts is involved in tissue growth, maintenance, and repair [250]. As the expression of GPCRs are extremely diverse and tissue-specific, these receptors are potential therapeutic targets in a broad array of diseases, which include fibrosis and cancer [237,250]. G proteins couple external stimuli with multiple intracellular signaling pathways. Depending on which G protein interacts with a specific GPCR, the outcomes of signaling are typically linked to characteristic extracellular stimuli. Further, the expression of GPCRs is often cell-specific in order to generate tissue-appropriate physiological responses [251].

GPCR signaling comprises multiple signaling cascades and pathways that have been extensively reviewed [250,252]. As examples of the complexity of GPCR signaling, we note that Gαs stimulates AC to increase production of the second messenger cAMP, which results in the activation of protein kinase A (PKA) and the exchange protein activated by cAMP (Epac) to enable productive downstream signaling [241]. Gαi/o is antagonistic to Gαs and suppresses AC, thereby decreasing production of cAMP [254]. Gαq/11 stimulates phospholipase C (PLC), which results in the cleavage of PIP_2_ and the production of diacylglycerol (DAG) and inositol-1,4,5-triphosphate (IP_3_). Subsequently, IP_3_ triggers the release of Ca^2+^ from the endoplasmic reticulum, and DAG activates protein kinase C (PKC) [255]. Gα12/13 activates the small GTPase Rho, which regulates cytoskeletal remodeling and cell growth [256]. In particular, GPCR signaling has been implicated in the development of fibrotic lesions in which different Gα subclasses inhibit or promote pro-fibrotic feedback signaling (Table 2) [223,250].

### 9.1. Gαi/o, Gαq/11, Gα12/13 Downstream Pathways Contribute to Fibrosis

GPCR expression can activate different Gα-protein subclasses through the same receptor [232]. Some of the GPCRs that play a role in promoting fibrosis include: protease-activated receptors [265,268,269], lysophospholipid receptors [259,260,261,262], adenosine receptors [257,258], angiotensin II [275,276], endothelin receptor A [277], serotonin receptors 2A and 2B [278], and sphingosine-1-phosphate receptor 1 [263]. As fibrosis can affect many different tissues, certain receptors are more likely to induce pro-fibrotic fibroblast activation based on the specificity of receptor expression in individual tissues [279].

**Figure 3:**
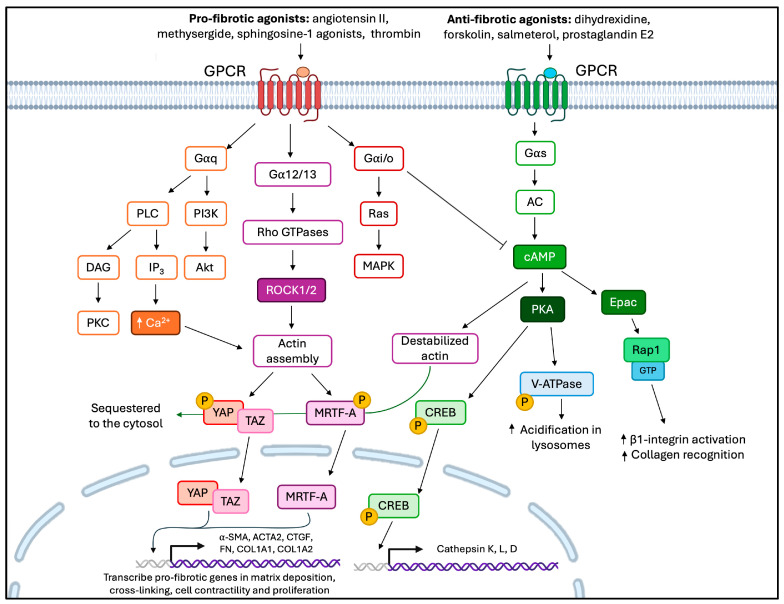
Different GPCR subclasses can contribute to or reduce fibrosis. Gαq/11 activates PI3K and PLC, leading to an increase in Ca^2+^ and the assembly of actin. Gα12/13 promotes actin assembly through Rho GTPases and ROCK1/2 signaling. Gαi/o inhibits cAMP and activates cell proliferation through the MAPK pathway. Agonists that activate GPCRs that are coupled to Gαq, Gα12/13, and Gαi/o G protein subclasses lead to fibrotic gene expression through YAP/TAZ and MRTF-A nuclear localization. Conversely, agonists that activate GPCRs that are coupled to Gαs produce cAMP. This promotes destabilized actin and sequesters YAP/TAZ and MRTF-A. cAMP signaling activates PKA and Epac. PKA canonically activates CREB, which promotes cathepsin gene expression and also phosphorylates v-ATPases, which acidifies lysosomes. cAMP stimulates Epac as well, which promotes Rap1 activity to increase collagen recognition and β1 integrin activation.

Certain GPCRs can activate Gαi/o, Gαq/11, or Gα12/13 subclasses [279], which we consider here in the context of generating pro-fibrotic signals. For example, the Gαi/o subclass suppresses cAMP production and can regulate Smad2/3 and extracellular signal-regulated kinase 1/2 (ERK1/2) phosphorylation [280], which in many tissues promotes fibrosis [261,262,280]. The Gαq/11 promotes fibrosis by increasing Ca^2+^ release from the endoplasmic reticulum (via IP_3_ receptors), which promotes collagen secretion and myofibroblast differentiation [13,281]. Pro-fibrotic agonists such as TGF-β1, angiotensin, and endothelin-1 activate robust Ca^2+^ signaling through PLC, which can enhance collagen I gene expression [282,283]. The Gα12/13 subclass stimulates ROCK signaling, which is required for myofibroblast differentiation [284]. Collectively, the activation of these pathways promotes the nuclear entry of YAP/TAZ and MRTF-A, which are important, pro-fibrotic mediators [230,285,286,287]. In addition, GPCR subclasses inhibit LATS1/2 kinase activity through Rho-dependent actin remodeling [231]. Further, as examples of the complex interplay between signaling cascades, GPCR agonists that activate Gαi/o, Gαq/11, Gα12/13 subclasses, increase the abundance of nuclear YAP, which sensitizes the responses of fibroblasts to TGF-β1 [287]. Notably, mechanosensitive processes that involve signaling through integrins and focal adhesion-mediated pathways also promote fibroblast activation through the same convergent pathways [217]. In sum, the GPCRs that selectively stimulate Gαi/o, Gαq/11, Gα12/13 subclasses also favor fibrogenesis and promote myofibroblast differentiation when they are dysregulated (Figure 3).

### 9.2. Gαs GPCR/cAMP Pathway Is Anti-Fibrotic

GPCRs that couple to the Gαs subclass activate AC, which subsequently elevates intracellular cAMP levels. Consistent with this notion, in cells stimulated with the AC activator forskolin, elevated cAMP levels block TGF-β1-induced myofibroblast differentiation and collagen synthesis [4,266,267,288]. Likewise, for cells in which GPCRs coupled to Gαs proteins were stimulated, the increased cAMP levels inhibited fibroblast proliferation, collagen production, myofibroblast differentiation, and actin cytoskeletal remodeling [5,266,267,288,289,290]. Further, in pulmonary fibroblasts, increasing cAMP levels with a Gαs GPCR agonist enhanced the degradation of collagen I by lysosomal cathepsin K and reduced ECM stiffness [242].

The second messenger cAMP has two main downstream effector proteins, PKA and Epac, each of which exhibits anti-fibrotic effects independently of the other (Figure 3) [241,291]. PKA is a classical cAMP effector that phosphorylates cAMP-response element-binding protein (CREB; at serine residue 133) and subsequently transits to the nucleus, where it associates with CREB-binding protein (CBP) [4,292,293]. Signaling through cAMP/PKA/CREB/CBP blocks TGF-β1-induced interaction of Smad3 with CBP, which is necessary for myofibroblast differentiation [292,294,295]. Activation of CREB/CBP increases the expression of Smad7, a negative regulator of Smad3 that inhibits collagen expression [296].

Interestingly, PKA agonists can inhibit collagen I expression and revert the phenotype of myofibroblasts to fibroblasts [291,297]. In kidney cells, cAMP mediates PKA activation that increases phosphorylation of the vacuolar-ATPase and subsequent endosomal acidification in kidney cells and astrocytes [298,299]. Additionally, CREB may be involved in modulating the expression of lysosomal cathepsins L and B, possibly enhancing collagen degradation [186,300]. Likewise, when cAMP/PKA signaling in osteoclasts is inhibited, cathepsin K processing and maturation are reduced, which highlights the role of cAMP in lysosomal degradation [274].

In pulmonary fibroblasts, a Gαs GPCR, the dopamine D1 receptor (DRD1), promotes cAMP downstream signaling, which is manifest as increased intracellular collagen degradation [242]. Gαs GPCR signaling also enhances extracellular cleavage, internalization, and lysosomal degradation of collagen I mediated by cathepsin K, and this effect persists even in cells treated with the pro-fibrotic growth factor, TGF-β1 [242,273]. In multiple cell types, Gαs GPCR/cAMP signaling enhances acidification in lysosomal compartments, which provides an acid-optimal environment for cathepsin activation [242,270,271,272]. Indeed, collagenolytic cathepsins can reduce collagen deposition in response to drug-induced fibrosis, which may be upregulated by CREB [23,68,70,186,301]. But in certain types of fibrotic lesions (pulmonary fibrosis), increased extracellular cathepsin K can activate fibroblasts and promote collagen synthesis, demonstrating the complexity of the formation of fibrosis [302].

**Figure 4:**
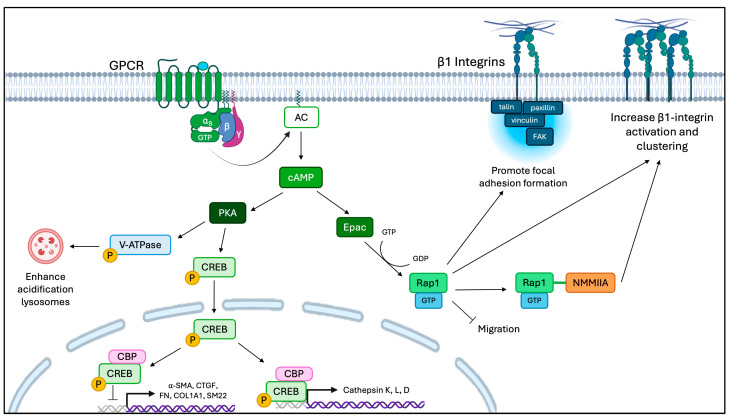
cAMP signaling and ECM interactions. Anti-fibrotic agonists that target Gas GPCRs activate AC, which produces cAMP from ATP. cAMP has two downstream effectors, PKA and Epac. PKA phosphorylates CREB, which transits to the nucleus and associates with CBP. CREB/CBP inhibits pro-fibrotic gene expression and increases cathepsin gene expression. PKA can also increase phosphorylation of v-ATPase and subsequent endosomal acidification. cAMP enhances acidification, which provides an acid optimal environment for cathepsin activation and increases collagen degradation. The other downstream effector Epac activates Rap1, which subsequently reduces migration, decreases proliferation, promotes focal adhesion formation, and interacts with NMMIIA to enhance β1 integrin-mediated collagen phagocytosis.

A separate effector protein that is activated by cAMP is the guanine exchange factor, Epac, which activates the small GTPases Rap1 and Rap2 [303,304]. When cAMP levels increase, Epac is rapidly activated and is redistributed in the cell to promote localized signaling processes [305]. Rap1 is a downstream effector of many pathways, including Ca^2+^-mediated signaling cascades that involve PLC and release of intracellular Ca^2+^ from the endoplasmic reticulum [306,307,308,309]. Rap1 is also an upstream regulator of Ca^2+^ signaling, which exemplifies the importance of “crosstalk” between these signaling platforms in different cell types [309]. In fibroblasts, Rap1 activation reduces migration, decreases proliferation, and interacts with NMMIIA to enhance β1 integrin-mediated collagen phagocytosis [48,310]. A direct link between Epac and collagen phagocytosis has yet to be determined, and an intriguing question is whether Epac increases collagen degradation in the phagocytic pathway by promoting Rap1 activation, which does enhance collagen phagocytosis (Figure 4) [48].

The second messenger cAMP can play a role in regulating focal adhesion function and signaling, as well as actin filament turnover through Rap1-GTP-mediated responses, but this conjecture has not yet been explored. Downregulation of Epac signaling is required for collagen fibrogenesis, as earlier data showed that during pro-fibrotic activation of fibroblasts in multiple tissues, Epac mRNA and protein expression were reduced [310]. Earlier observations in cardiac fibroblasts observed a role of Epac inhibition in collagen synthesis and myofibroblast differentiation [310,311] and suggest a possible anti-fibrotic therapeutic purpose in this pathway. To discern the differential effects of cAMP effectors, cAMP derivatives that selectively activate Epac (8-Me-cAMP) and PKA (N6-cAMP) have been used [241]. Further, cAMP signaling, particularly through Epac, reduces nuclear localization of the pro-fibrotic transcription factors YAP/TAZ and MRTF-A [5,231,273,289,312,313,314]. Since cAMP signaling is a fundamental system for normal cellular function, it is tightly regulated by PDEs, ACs, and by crosstalk with other second messenger systems.

The concentration of cAMP in the cytoplasm of cells is dependent on the abundance, location, and activity of membrane-bound and soluble forms of adenylyl cyclase, and on the rate of cAMP degradation by PDEs [241,245,315]. ACs are the only enzymes that synthesize cAMP, and ACs can integrate signals received from Gαs GPCRs to convert adenosine triphosphate into cAMP [316]. In mammals, there are nine transmembrane isoforms of ACs (AC1-AC9) and one soluble AC that displays varying expression levels in different cells and tissues [317]. Both cardiac and pulmonary fibroblasts mainly express AC3, AC5, and AC6 isoforms, but other isoforms are expressed depending on their subcellular localization [283,318,319]. In pulmonary fibroblasts, overexpression of AC6 reduced the expression of type I collagen and increased the expression of MMP-2 [320], a gelatinase that can reduce the abundance of extracellular collagen. Notably, in comparison to fibroblasts, myofibroblasts produce less cAMP and exhibit decreased expression of AC isoforms and increased expression of PDEs [292].

PDEs convert cAMP into 5′-adenosine monophosphate. There are multiple PDE isoforms in fibroblasts, which include PDE1A [321], PDE4B [247], PDE5 [322], PDE7 [323], and PDE8 [323]. PDE1A belongs to the Ca^2+^/calmodulin-stimulated PDE1 family and promotes ECM synthesis and myofibroblast differentiation in cardiac fibroblasts [323], an observation that underpins the importance of the relative abundance of cAMP in the control of pro-fibrotic processes. The use of experimental approaches that enhance cAMP levels in fibroblasts (e.g., agonists for Gαs GPCRs, PDE inhibitors, PKA enhancers, and Epac activators) provides potential therapeutic targets for clinical management of fibrotic lesions [223,241]. The crosstalk of cAMP with Ca^2+^-dependent signaling pathways contributes to the complexities of understanding how fibrosis is promoted [324,325]. Since Ca^2+^ signaling involves multiple pathways [326], we will provide detailed information on TRPV4 and focus on Ca^2+^ flux through TRPV4 channels because of its implications in fibrosis below.

## 10. TRPV4 Is a Ca^2+^ Permeable, Mechanosensing Channel That Promotes Fibrosis

The mammalian Transient Receptor Potential (TRP) channels are conserved, integral membrane proteins that are grouped into six subfamilies based on sequence homology [327]. In particular, the Vanilloid TRP subfamily has six members: TRPV1–4 are non-selective cation conducting pores, while TRPV5 and TRPV6 are highly Ca^2+^ selective [327]. TRPV4 is broadly expressed in several different tissues and by multiple cell types, notably in the peripheral nervous system (root ganglia, trigeminal ganglia, hippocampal pyramidal neurons, and retinal ganglion cells) [328], and importantly, by fibroblasts (Table 3) [61]. Multiple stimuli can activate TRPV4, including heat, osmotic pressure changes, chemical stimulation, and mechanical stress [329,330,331,332,333]. Activation of TRPV4 in fibrotic tissues promotes fibroblast differentiation into contractile myofibroblasts and enhances collagen deposition [208,329,334].

Two recent reviews have considered several mechanisms that link TRPV4 mechanotransduction and Ca^2+^ influx in the context of fibrosis [21,208]. Here, we will focus on Ca^2+^ conductance and mechanosensing by TRPV4, as it has been linked to the pathophysiology of multiple diseases, including fibrosis, cancer, and neurodegenerative diseases [208,329].

Fibrotic disease progression can be promoted by dysregulated TRPV4 signaling. TRPV4-induced Ca^2+^ influx is required for TGF-β1-induced myofibroblast differentiation in numerous tissues, including the heart, lung, airways, and skin [208,329,334]. In mice subjected to bleomycin-induced fibrosis, TRPV4 expression is upregulated [329]. In TRPV4-deficient mice, the animals are protected from bleomycin-induced pulmonary fibrosis and exhibit reduced myofibroblast differentiation. These processes are reversed when TRPV4 is expressed [329]. Likewise, following suppression of TRPV4 function by chemical antagonists, siRNA-mediated knockdown, or loss-of-function genetic manipulation, TGF-β1-mediated type 1 collagen and fibronectin production are reduced, and force-induced cellular contractility, epithelial-to-mesenchymal transition, and α-SMA expression and incorporation into stress fibers are all inhibited [208,354,357,362].

**Figure 5:**
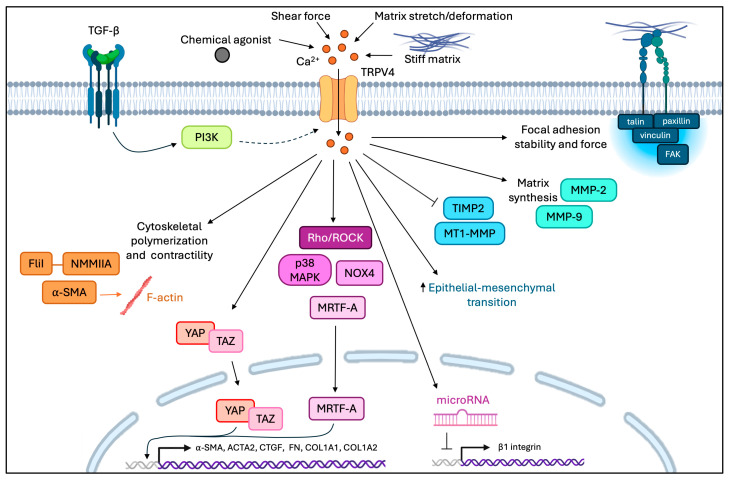
TRPV4 activation regulates matrix remodeling and activates pro-fibrotic pathways. TRPV4 activation can regulate matrix remodeling through TGF-β1 or independently. TRPV4 regulates mechanosensitive transcription factors YAP/TAZ, MRTF-A, matrix synthesis and turnover, focal adhesion stability, EMT, microRNA inhibition of β1 integrin cytoskeletal polymerization, and contractility. Adapted from Ji and McCulloch, 2021 [244]. The interaction between PI3K and TRPV4 is denoted by a dashed arrow, as it has not been defined by supported data.

TGF-β1, which is one of the most potent pro-fibrotic agonists that have been described, activates phosphoinositide 3-kinase (PI3K), NADPH oxidase 4 (NOX4), ROCK, and p38 MAPK during fibroblast differentiation and increases nuclear localization of MRTF-A and YAP/TAZ (Figure 5) [208,334,354]. In cells treated with the TRPV4 inhibitor RN1734, the activation of Rho, p38 MAPK, and MRTF-A nuclear localization was all inhibited [334]. Interestingly, PI3K was not altered by TRPV4 inhibition, indicating that PI3K is upstream of TRPV4 and regulates TRPV4 in response to TGF-β1 in fibroblasts [334].

TRPV4 activation enhances matrix synthesis and strongly increases the expression of α-SMA [334]. Evidently, TRPV4 regulates the pro-fibrotic functions of TGF-β1 by modulating both the non-canonical SMAD-independent and the SMAD-dependent downstream pathways [329,334,357,363]. Independent of TGF-β1, TRPV4 independently upregulates the expression of α-SMA, the formation of highly aligned collagen fibrils, and the activation of MRTF-A, as shown in cultured cells treated with the small molecule TRPV4 agonists GSK1016790A and 4α-PDD (Figure 5) [208,364]. In response to increased matrix stiffness and TGF-β1, the expression of TRPV4 is needed for nuclear translocation of YAP/TAZ, myofibroblast differentiation, and for the regulation of epithelial-to-mesenchymal transition [357].

In matrix mechanosensing, TRPV4 can impact ECM remodeling, which is dependent on Ca^2+^ conductance through TRPV4 channels [329,337,365]. TRPV4 is enriched in focal adhesions and is activated by mechanical force transfer from the β1 integrin cytoplasmic binding protein, CD98 [366]. Higher TRPV4 expression is associated with reduced β1 integrin abundance, adhesion to collagen, focal adhesion size, alignment, and the compaction of extracellular collagen fibers [367]. The expression of TRPV4 enhances microRNAs that promote downregulation of β1 integrin mRNA, which directly impacts the ability of cells to recognize and bind tightly to the underlying ECM [367]. Subsequently, after activation, TRPV4 initiates Ca^2+^ oscillations that trigger signaling, which are involved in multiple cellular processes like tissue repair and ECM remodeling [368]. In response to pro-fibrotic stimulation, elevations of Ca^2+^ are solely dependent on TRPV4 and are not affected by TGF-β1 [337]. In collagen remodeling, TRPV4-mediated Ca^2+^ influx regulates the functions of the actin-binding protein Flil and NMMIIA to enable the generation of cell extensions and enhanced contractility [61]. TRPV4-dependent Ca^2+^ influx is also associated with the collagen receptor DDR1, which influences processes that mediate collagen compaction and alignment [214]. In human mesenchymal stem cells, TRPV4 Ca^2+^ signaling is critical for the initial stages of collagen matrix assembly and can regulate force generation at cell–matrix adhesions [364].

TRPV4 activity influences the abundance of matrix-degrading MMPs in specific cell types and microenvironments. For example, in human respiratory epithelial cells, Gi/o GPCRs, PLC, and PI3K activate TRPV4 Ca^2+^ influx, which subsequently promotes MAPK-mediated MMP-1 production and secretion [338]. In whole mouse lungs, TRPV4 activation increases the abundance and activation of the gelatinases, MMP-2 and MMP-9, while reducing the expression of the MMP inhibitor, tissue inhibitor of metalloproteinases 2 (TIMP-2) and MT1-MMP [369]. MMP-2 and MMP-9 associate and cleave β1 integrins in colorectal carcinoma and in corneal epithelial cells [370,371]. TRPV4 increases the expression of MMP2 and MMP9, which reduces β1 integrin expression [369] and possibly affects the recognition of collagen fibrils at focal adhesions. TRPV4 also reduces MT1-MMP expression [369], which may affect collagen phagocytosis and the intracellular degradation of collagen. Indeed, MT1-MMP plays a crucial role in facilitating collagen phagocytosis by preparing collagen fibrils for subsequent internalization [43]. TRPV4-induced MT1-MMP downregulation has not been explored in the context of collagen phagocytosis but may contribute to reduced intracellular collagen degradation. In summary, TRPV4 is evidently involved in collagen remodeling. When dysregulated, TRPV4 contributes to the development and progression of fibrotic lesions, thereby indicating that TRPV4 is an attractive target for therapeutic inhibition.

## 11. Gαs GPCR and TRPV4 Signaling Are Interconnected Through the Second Messengers, cAMP and Ca^2+^

As described above, impaired collagen remodeling is one of the main characteristics of fibrosis, in which the accumulation of poorly organized, stiff collagen in the ECM is favored. In order to reduce the “burden” of excessive fibrillar collagen in the ECM, it will be important to discover which pathways specifically enhance intracellular collagen degradation. One pathway that is being explored as a potential therapeutic for reducing fibrillar collagen in the ECM is the Gαs GPCR-mediated pathway, as it is clearly involved in resolving fibrotic lesions through the involvement of cAMP downstream effectors [186,241,314,320]. The cAMP effector Epac mediates activation of Rap1, which interacts with NMMIIA to enhance collagen internalization and degradation in the phagocytic pathway [48,241]. Gelsolin also interacts with NMMIIA; however, this interaction is Ca^2+^-dependent [10], which may be mediated through TRPV4 or possibly by other Ca^2+^-permeable channels or from store-operated Ca^2+^ entry (Box 4).

Box 4 **Ca^2+^ signaling involves external and internal stores.**

Many plasma membrane, Ca^2+^-permeable channels control Ca^2+^ influx from the external environment in response to diverse stimuli, including mechanical forces, decreased cytosolic Ca^2+^, membrane depolarization, extracellular agonists, intracellular messengers, and depletion of intracellular stores [200]. Many stimuli activate the release of Ca^2+^ from intracellular stores (through PLC). Gαq/11 GPCRs generate IP_3_ [110,200], which binds to its cognate receptor on the endoplasmic reticulum to trigger Ca^2+^ release and oscillations [110,200]. Ca^2+^ controls Ca^2+^ release from intracellular stores, which are also affected by a large group of messengers (e.g., IP_3_, sphingosine-1-phosphate) [200].

In human pulmonary fibroblasts, Ca^2+^ oscillations contribute to the pro-fibrotic effects of TGF-β1, while prostaglandin E_2_ (PGE_2_), EP_2_ and EP_4_ receptors (which regulate AC), disrupt pro-fibrotic signaling through cAMP/PKA [372]. TGF-β1 triggers Ca^2+^ oscillations in human pulmonary fibroblasts [282,372] and in human ventricular fibroblasts, TRPV4-dependent Ca^2+^ influx mediated TGF-β1-induced myofibroblast differentiation: in these experiments, TRPV4 was the only TRP channel upregulated at mRNA and protein levels [373].

Ca^2+^ oscillations can modulate the expression of ECM genes in a frequency-dependent manner, either through TGF-β1 or independent of TGF-β1 [182,282]. The Ca^2+^ oscillations that are modulated by TGF-β1 are generated by the activation of Ca^2+^/calmodulin-dependent protein kinase-II (CaMK-II) and by TRPV4-activated pro-fibrotic signaling processes [373,374,375]. The anti-fibrotic effect of PGE_2_ is thought to be mainly explained by the interference of the EP_2_ receptor with Ca^2+^ signals. These signals inhibit CaMK-II and Akt activation in TGF-β1-treated pulmonary fibroblasts [372].

In certain cell systems, Ca^2+^ signaling directly regulates cAMP by modulating AC and PDE function [283,315,317,321]. For example, in osteocyte mechanotransduction, TRPV4- mediated Ca^2+^ influx disrupts AC6 function [376]. It is unknown whether this process also occurs in fibroblasts, but conceivably, TRPV4 Ca^2+^ signaling can reduce cAMP levels by adjusting the catalytic activities of ACs and PDEs. Ca^2+^ inhibits AC5 and AC6 isoforms and reduces cAMP generation in neuronal cells and cardiac myocytes [377,378,379]. Ca^2+^ may inhibit the production of cAMP by inhibiting AC and thereby reducing collagen degradation in fibrotic lesions. Likewise, Ca^2+^ can regulate cAMP by promoting the activity of PDE1 and PDE4, a process which depletes cAMP from cells [245,249,321,362,380]. In cardiac fibroblasts, PDE1A activity reduces cAMP-Epac-Rap1 signaling, which leads to increased myofibroblast formation and ECM synthesis [321]. Similarly, PDE4 promotes signaling through the MAPK, p38α, which generates a pro-fibrotic response in skin and lung fibroblasts [380].

**Figure 6:**
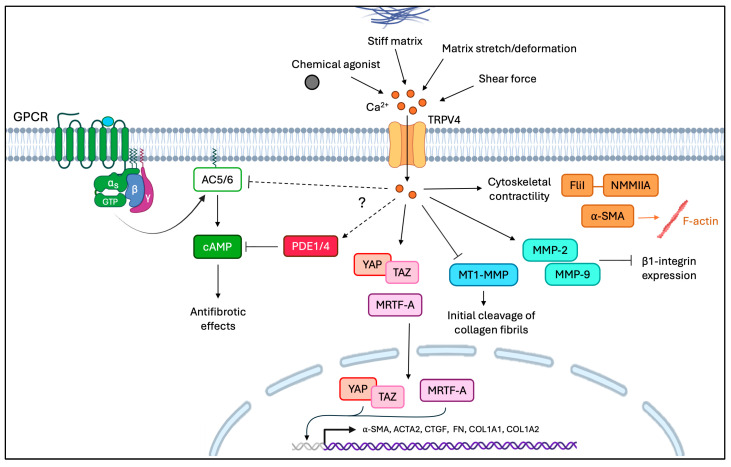
Interplay between cAMP and TRPV4 signaling systems in the context of ECM remodeling. TRPV4-mediated Ca^2+^ influx promotes nuclear localization of YAP/TAZ and MRTF-A, enhancing matrix synthesis and cytoskeletal contractility. TRPV4 Ca^2+^ influx plays a role in ECM remodeling. TRPV4 enhances MMP-2 and MMP-9, but inhibits MT1-MMP. TRPV4 Ca^2+^ influx enhances calcium-activated PDE1/4, which decreases cAMP levels. TRPV4 Ca^2+^ influx also modulates AC5/6, which reduces cAMP generation in certain cell types. The interaction between TRPV4 and AC5/6 or PDE1/4 is denoted by a dashed arrow, as it has not been defined by supported data.

In a therapeutic context, PDE4 inhibitors in combination with PGE_2_ agonists can alleviate organ fibrosis by enhancing the “dedifferentiation” of myofibroblasts into fibroblasts [380]. Likewise, PDE1 and PDE4 inhibitors increase cAMP to promote an anti-fibrotic response [247,249,323]. TRPV4-mediated Ca^2+^ influx may also be involved in activating PDEs and inhibiting AC5/6, which decreases cAMP and promotes pro-fibrotic responses (Figure 6). Notably, Ca^2+^ signaling not only upregulates pro-fibrotic responses: increased Ca^2+^ signaling can also induce AC3 to produce cAMP and thereby attenuate collagen synthesis in cardiac fibroblasts [283]. Further, cAMP can modulate Ca^2+^ influx by reducing Ca^2+^ channel activities in vascular smooth muscle cells, although the potential clinical implications for the management of fibrosis are not understood [381]. Collectively, the complex interactions between cAMP and Ca^2+^ signaling may be important contributing mechanisms that alter the balance between collagen synthesis and degradation. Further, in-depth examination of the interplay between TRPV4 and Gαs GPCR cAMP pathways may provide new insights into potential anti-fibrotic therapeutics.

## 12. Conclusions and Future Perspectives

Small perturbations in TGF-β1, TRPV4, and Ca^2+^-mediated signaling can create positive feedback loops that promote fibrosis and drive aberrant collagen remodeling. Accordingly, understanding the fundamental mechanisms that drive tissue-specific fibrotic lesions by analyzing relevant signaling pathways may be useful in developing rational treatment options. We have reviewed above which GPCR pathways enhance or reduce fibrosis progression, based on the specific Gα proteins that are activated. Specifically, we focused on Gαs GPCRs, which generate cAMP and signal through PKA and Epac to elicit an anti-fibrotic response. Both of these cAMP downstream pathways (i.e., Epac and PKA) decrease the activation of pro-fibrotic signaling systems by decreasing myofibroblast activation, enhancing collagen degradation, and reducing pro-fibrotic gene expression [3,242,295,306,310,380]. An important gap in our understanding of pro-fibrotic processes is that the role of cAMP signaling in regulating focal adhesions and cytoskeletal dynamics through Rap1 is not defined. In the context of collagen remodeling, focusing on Gαs GPCR pathways and their relationships with pro-fibrotic signaling pathways would be beneficial to provide more focused targets for potential treatments.

We considered here the role of TRPV4 in collagen remodeling and mechanosensing. Notably, TRPV4 function and activation are integrated with β1 integrin-mediated adhesion to collagen. But with stiff matrices, TRPV4 becomes dysfunctional and promotes pro-fibrotic signaling [367]. Understanding the role of TRPV4 Ca^2+^ signaling in fibrosis is complicated because it is also involved in a large cadre of processes, including wound healing, cytoskeletal remodeling, epithelial–mesenchymal transformation, and matrix synthesis and turnover [208]. Currently, while it is not known whether TRPV4 influences collagen internalization directly, recent data indicates that TRPV4 expression decreases β1 integrin expression through microRNAs and reduces MT1-MMP expression [367,369].

Maintaining a tight balance between collagen synthesis and degradation is important for healthy organ structure and function. Accordingly, we considered here how Ca^2+^ and cAMP signaling are connected in physiological collagen remodeling and what processes occur when these pathways are disrupted in fibrosis. Based on current analyses, the interplay between TRPV4 and GPCR is not fully defined. Although their downstream pathways and associated second messengers exhibit extensive crosstalk, our understanding of the underlying regulatory mechanisms, compensatory systems, and interactions is incomplete. For example, in osteocytes, Ca^2+^ flux through TRPV4 inactivates ACs [376], but it is unknown if this same effect also occurs in fibroblasts. Ca^2+^ signaling is known to regulate PDEs that are expressed in fibroblasts [245,372,380], but the role of TRPV4 channels in these processes is not understood. Using mouse models with fibroblast-specific deletion of TRPV4 fibroblasts could help to distinguish whether Ca^2+^ signaling in PDE regulation is mediated by TRPV4 or other channels. More defined data sets describing how TRPV4-mediated Ca^2+^ signaling regulates AC and PDE function are needed to understand the functional interplay between cAMP and TRPV4 in fibrotic lesions.

Exploring whether Gαs GPCR agonists can rescue TRPV4-driven pro-fibrotic phenotypes is functionally relevant in new treatments for fibrosis and for preservation of physiological collagen remodeling. Since the Gαs GPCR pathway increases intracellular collagen degradation and decreases myofibroblast differentiation in response to TGF-β1 signals [4,242,289], we anticipate cAMP stimulators may reverse TRPV4-mediated, pro-fibrotic collagen remodeling. In the context of ECM health, defining the relationship between TRPV4 and Gαs GPCRs signaling provides an opportunity to discover new drug targets for the development of new therapies for clinical management of fibrosis.

## Figures and Tables

**Table 1:** Therapeutic drugs that contribute to fibrosis.

Drug of Interest	Main Therapeutic Function of Drug	Affected Tissues or Cells	Pro-Fibrotic Signaling Systems Involved	Fibrotic Mechanism	Ref.
Isoniazid, Rifampicin, Ethambutol	Antituberculosis, antimicrobial drug	Liver	- Oxidative stress - Mitochondrial damage - Production of reactive nitrite and oxygen species - Endoplasmic reticulum stress - Cell death - DNA damage - Increase calcium levels - Enhance immune infiltration	Indirectly fibrotic	[81,82,83]
Sulfasalazine	Antibiotic used to treat ulcerative colitis and Crohn’s disease	Heart	- Activates the Akt signal pathway, exacerbating angiotensin II-induced cardiac remodeling	Directly fibrotic	[84]
		Liver	- Prevents lipid peroxidation - Blocks the accumulation of collagen in the liver - Decreases expression of TGF-β	Anti-fibrotic	[85]
		Lung	- Induces interstitial fibrosis - Induces lung toxicity through a delayed allergic mechanism	Indirectly fibrotic	[86]
Alemtuzumab	Anti-CD52 monoclonal antibody targets the destruction of CD52+ cells in blood cancers and multiple sclerosis	Lymphocytes	- Autoimmune storm - Chronic antibody-mediated rejection and myelofibrosis	Indirectly fibrotic	[87,88]
Bevacizumab	Anti-vascular endothelial growth factor monoclonal antibody blocking angiogenesis in cancer	Liver	- Downregulates expression of α-SMA and TGF-β1 - Blocks activation and proliferation of hepatic stellate cells	Anti-fibrotic	[89]
		Retinal pigment epithelial cells	- Increased gene and protein expression of IL-1β, IL-6, IL-8, and TNF-α	Directly fibrotic	[90,91]
		Umbilical vein endothelial cells	- Upregulation of TGF-β, MMP-2, connective tissue growth factor, fibroblast growth factor	Directly fibrotic	[92]
Cetuximab	Anti-epidermal growth factor Receptor monoclonal antibody to treat metastatic cancer	Liver	- Reduces inflammatory cell infiltration - Reduces expression of NF-κB, iNOS, IL-6, TNF-α, and TGF-β. - Could lead to liver toxicity	Mainly anti-fibrotic	[93]
		Epidural space	- Decreases fibroblast numbers and density	Anti-fibrotic	[94]
		Lung	- Pulmonary toxicity is rare - Increases pulmonary infiltrates	Indirectly fibrotic	[95]
Trastuzumab	HER2-monoclonal antibody to treat cancer	Heart	- Increases protein expression of CD74, p-STAT1, Bax, Caspase-3, IFN-γ and TNF-α - Induces cardiotoxicity	Indirectly fibrotic	[96]
TNF-α blockers	TNF-α monoclonal antibody to treat rheumatoid arthritis, Crohn’s disease	Lung	- Progression of interstitial lung disease - Diffuse alveolar damage - Alters tissue repair - Varies between patients	Indirectly fibrotic	[97,98,99]
Amiodarone	Anti-arrhythmic	Lung	- Promotes intense collagen deposition - Disturbs nitric oxide synthase enzymes - Increases α-SMA and p-STAT3 expression - Upregulates TGF -β1/PI3K/Akt1-p axis - Induces pulmonary toxicity	Directly fibrotic	[100,101]
		Liver	- Induces lipid accumulation associated with ER stress and apoptosis - Increases keratin-18 fragments in serums - Promotes the development of steatohepatitis, liver fibrosis, and cirrhosis	Directly fibrotic	[102]
β-blockers	β-adrenergic receptor agonists that treat cardiovascular conditions like high blood pressure and heart arrhythmias	Heart	- G protein-independent and GPCR kinase 5/β-arrestin2-dependent - pathway inducing cardiac fibrosis	Directly fibrotic	[103,104]
Flecainide	Antiarrhythmic medication that blocks cardiac sodium channels	Lung	- Promotes diffuse infiltrative lung disease with lymphocytic alveolitis - High affinity for concentrating in lung tissue - Cell-mediated immunologic reaction	Indirectly fibrotic	[105,106]
Hydrochlorothiazide	Thiazide diuretic for the treatment of hypertension	Heart	- Decrease myocardial ROCK activation - Decrease expression of pro-remodeling, pro-fibrotic, and pro-oxidative genes	Anti-fibrotic	[107]
		Retroperitoneal space	- TGF-β/Smads cascade-mediated enhancement of myofibroblast proliferation - Overproduction of ECM components	Indirectly fibrotic	[108]
Procainamide	Antiarrhythmic medication that blocks voltage sodium channels	Lung	- Associated with the release of oxygen radicals and some mediators such as tumor necrosis factor-α, TGF-β, platelet-derived growth factor-B, and insulin-like growth factor-1, endothelin-1, and interleukins 1, 4, 8, and 13.	Directly fibrotic	[109,110]
Tocainide	An antiarrhythmic that is structurally related to lignocaine, which blocks voltage-gated sodium channels	Lung	- Irreversible pneumonitis, pulmonary toxicity, and bilateral interstitial fibrosis - Mechanism is not fully known, but may be pro-inflammatory and a hypersensitivity reaction - Was taken off the market	Indirectly fibrotic	[111]
Azathioprine	An immunosuppressant used to treat inflammation	Lung	- Inhibit Band T-lymphocyte proliferation - Severe and infrequent adverse effect	Indirectly fibrotic	[112]
BCNU (Carmustine)	Chemotherapy	Lung	- Increase oxidative stress - Directly damage pneumocyte and endothelial cells - Increased expression of platelet-derived growth factor-b and insulin-like growth factor-1 on alveolar macrophages and type II pneumocytes - Decreased expression of TGF-β1 and cyclooxygenase-2	Directly Fibrotic	[113,114]
Bleomycin	Antimitotic drug used to treat cancer	Lung	- Increases proliferation of fibroblasts, cell damage, and infiltration of immune cells - Increases interlukin-11 which induces p-STAT3 - Chronic release of pro-inflammatory and pro-fibrotic molecules - Oxidative damage	Directly and Indirectly fibrotic	[115,116,117]
Busulfan	DNA-alkylating antineoplastic for cancer	Lung	- DNA alkylation and cellular senescence - Promotes a hyper-inflammatory state - Increase collagen deposition - Increase a-SMA, TNF-α, caspase 3, NOX-4 - Decrease cyclooxygenase-2	Directly fibrotic	[118,119]
		Liver	- DNA alkylation and cellular senescence - Induce liver inflammation, NLRP3 activation - Endoplasmic reticulum stress	Indirectly fibrotic	[120,121]
Chlorambucil	DNA-alkylating antineoplastic agent to treat cancers	Lung	- Drug-induced pneumonitis - Interstitial round cell infiltrates - Proliferation of type II alveolar lining cells	Indirectly fibrotic	[122,123,124]
Cyclophosphamide	DNA-alkylating antineoplastic agent to treat cancers	Lung	- Dosage-dependent effect - Increase TNF-α and IL-6 cytokines - Promotes inflammation through JAK-2/STAT-3 axis, modulating autophagic and apoptotic signals	Indirectly fibrotic	[125,126]
		Heart	- Associated with apoptosis, inflammation, endothelial dysfunction, calcium dysregulation, endoplasmic reticulum damage, and mitochondrial damage. - Crosstalk of Nrf2/ARE, Akt/GSK3β/NFAT/calcineurin, p53/p38MAPK, NF-kB/TLR-4, and phospholamban/SERCA-2a signaling pathway.	Indirectly fibrotic	[127,128]
		Liver	- Oxidative stress, inflammation, apoptosis, and fibrosis via modulation of Nrf2, NF-κB p65, caspase-3, TGF-β1, and associated biochemical status	Indirectly fibrotic	[129]
		Kidney	- Inhibits the proliferation of mesangial cells by modulating cell cycle regulators - Significantly alleviates the severity of renal fibrosis	Anti-fibrotic	[130]
Cytarabine	Antimetabolite chemotherapy that interferes with DNA synthesis and repair	Lung	- Lung infiltrates - At high doses, cytarabine induced noncardiogenic pulmonary edema	Indirectly fibrotic	[131]
		Heart	- Inflammatory cell infiltration and fibrosis - Induces mitochondrial dysfunction and endoplasmic reticulum stress	Indirectly fibrotic	[132]
Docetaxel	Antimitotic chemotherapy disrupting the microtubule dynamics	Lung	- Increases the prevalence of interstitial pneumonitis, cardiogenic pulmonary edema, pleural effusions, and peripheral edema - Could be due to a hypersensitivity reaction - Cellular damage, immune activation, and oxidative stress	Indirectly fibrotic	[133,134]
Erlotinib	Epidermal growth factor receptor tyrosine kinase inhibitor to treat cancer	Lung	- Infrequent induction of interstitial lung disease - No published randomized controlled trials because of the rarity of presentation	Indirectly fibrotic	[135,136,137]
Etoposide	Chemotherapeutic that inhibits topoisomerase II, leading to irreversible DNA damage in cancer cells	Lung	- General cytotoxicity - Abnormal epithelial–mesenchymal interactions - Induction of apoptosis	Indirectly fibrotic	[138,139]
Fludarabine	Chemotherapy agent that is a purine analog antimetabolite inducing cell death	Lung Bone Marrow	- Rare and serious side effect that induces fibrosis - Not fully understood, but it is thought to involve immune-mediated hypersensitivity - Cytotoxic actions	Indirectly fibrotic	[140,141]
Flutamide	Non-steroidal antiandrogen antagonist used to treat cancer	Liver	- Induces idiosyncratic liver injury - Increases caspase-1 activity and production of IL-1β - Promotes the release of damage-associated molecular patterns that activate inflammasomes	Indirectly fibrotic	[142,143]
		Lung	- Not fully understood, but may be due to an increase in oxidative stress, immune-mediated toxicity,	Indirectly fibrotic	[144,145]
Melphalan	Alkylating chemotherapy drug that interferes with DNA and RNA synthesis used to treat cancer	Lung	- Cytotoxic agent that directly damages lung tissues	Indirectly fibrotic	[146]
Methotrexate	Folic acid analog used to treat dermatological diseases	Lung	- Promote epithelial–mesenchymal transition and tumor necrosis factor production - Induces pulmonary toxicity - Induces IL-6/STAT3 pathway	Directly fibrotic	[147,148,149]
		Liver	- Oxidative stress, impaired mitochondrial respiration, and endoplasmic reticulum stress - Increases in plasma alanine aminotransferase, cytokeratin-18, malondialdehyde levels, TGF-β, YAP1 - Increase ECM deposition	Directly fibrotic	[150,151,152,153]
Nitrosoureas (i.e., Methyl-CCNU)	Alkylating nitrosourea chemotherapy	Kidney and Lung	- Elevation of blood urea nitrogen - Pulmonary toxicity is rare - Cell damage	Indirectly fibrotic	[154,155,156]
Vinblastine	Anticancer drug binds to tubulin and arrest cells in M-phase	Lung	- Diffuse interstitial pulmonary infiltrates - Produce pulmonary toxicity	Indirectly fibrotic	[157,158,159]
		Heart	- Induces myocardial damage and necroptosis.	Indirectly fibrotic	[160]
Bromocriptine, Cabergoline	Dopamine-2 receptor agonists that inhibits prolactin secretion	Lung, Brain Heart	- Uncommon adverse effect that induces pulmonary fibrosis at a low dose - Possibly, cell shrinkage causes enlargement of the extracellular and perivascular spaces - Increase fibrous tissue content - Prevalence of cardiac valve fibrosis	Directly fibrotic	[161,162,163,164]
Carbamazepine	Anticonvulsant medication that blocks voltage-gated sodium channels and is used to treat epilepsy	Liver	- Novel hepato-proliferative effect by activation of the mTOR pathway in mice - Induces hepatotoxicity - Has potential to facilitate liver regeneration	Both indirectly fibrotic and antifibrotic	[165,166,167]
		Lung	- Upregulates inflammatory genes and cell proliferation (IL6, IL1A, and RNU22) - Downregulates tissue development and ECM receptor interaction - Pulmonary toxicity	Directly fibrotic	[168,169]
Methysergide	Ergot alkaloid pro-drug; serotonin antagonist for migraine treatment	Heart	- Mitral and aortic valvular fibrosis and dysfunction - Agonist for the serotonin receptor on the heart connective tissue - Fibroblast proliferation and differentiation into myofibroblasts	Directly fibrotic	[170,171,172]
		Lung, Retroperitoneal	- Promotes inflammatory response and cytotoxicity - Pleural thickening - Activation of fibroblasts - Overproduction of ECM	Directly fibrotic	[173,174,175]
Phenytoin	Anticonvulsant drug to prevent seizures	Gingiva, Liver and Lung	- Hypersensitivity reaction - Induces the proliferation and differentiation of myofibroblasts - Increases collagen production - Inhibits cathepsin L - Increases IL-6, IL-8 and α-SMA - Exacerbates the tissue turnover	Directly fibrotic	[176,177,178,179]
Nifedipine	Antihypertensive	Liver	- Induced hepatitis it is a rare occurrence - Prevents hepatic fibrosis	Anti-fibrotic and indirectly fibrotic	[180,181]
		Lung	- Disrupts of Ca2+ signaling in fibroblasts and prevents bleomycin pro-fibrotic responses	Anti-fibrotic	[182]
		Heart	- Downregulates NOX4-derived reactive oxygen generation - Suppresses ERK1/2 and JNK signaling pathways - Inhibits cardiac fibroblast proliferation and differentiation	Anti-fibrotic	[183]
		Gingiva	- Gingival overgrowth - Reduces intracellular Ca2+ concentration - Impaired collagen breakdown - Increases collagen, fibronectin expression, and fibroblast proliferation	Directly fibrotic	[184,185]
Cyclosporine A	Immunosuppressant that inhibits calcineurin and T cells	Kidney, Gingival Connective Tissues	- TGF-β1 secretion - ECM accumulation - Promote epithelial–mesenchymal transition - May reduce collagen degradation - Inhibition of CREB	Directly fibrotic	[186,187,188,189]
Nitrofurantoin	Antibiotic to treat urinary tract infections	Lung, Connective Tissues	- Promotes activation of proto-myofibroblast cells with enlarged and less rounded morphology - Increases fibroblast motility - Supports ECM organization and induces pro-fibrotic signaling - Increases ECM stiffness and fibrotic progression - Releases inflammatory cytokines	Directly fibrotic	[190,191,192]
Pemetrexed	Chemotherapy drug that is a multitargeted antifolate Antimetabolite	Lung	- Damage to alveolar epithelial cells - Immune-mediated hypersensitivity reaction - Pulmonary toxicity	Indirectly fibrotic	[193,194,195]
Gemcitabine	Chemotherapy drug that is a pyrimidine antimetabolite that disrupts DNA synthesis	Lung	- Cytotoxicity - Uncontrolled inflammatory and repair process - Not fully understood	Indirectly Fibrotic	[196,197,198]
Doxorubicin	Chemotherapeutic, anthracycline antibiotic	Heart, Liver, Lung	- Induces inflammation, oxidative stress, and cellular senescence - Increases epithelial-to-mesenchymal transition - Accumulation of reactive oxygen species - Decrease autophagy	Directly fibrotic	[199,200,201,202,203]

**Table 2:** Involvement of GPCR sub-classes in fibrosis.

G Protein Subclass	Receptor Examples	Signaling Systems	Physiological Roles	Implications in Fibrosis	Ref.
Gαi/o	Apelin, AT1R, AT2R, A1-AR, A3-AR, CB1, CB2, C5aR, CCR2/5, CXCR4, S1PR1, LPAR1, M2, Fz2, SMO, Y1-R	Inhibition of adenylyl cyclase. Promotes Smad2/3 and ERK1/2 phosphorylation.	Necessary for tissue repair. Promotes proliferation, myofibroblast differentiation, and ECM synthesis.	Suppresses cAMP production, which leads to excessive ECM production.	[250,257,258,259,260,261,262,263,264,265,266,267]
Gαq/11	AT1R, ETAR, ETBR, LPARs, PAR1, PAR2, S1PR2, S1PR3, M1/3, 5-HT2A, 5-HT2B, ADRA1A, AVPR1A, GPR55	Activation of PLC leads to the production of DAG and IP_3_. DAG -> PKC Increases Ca^2+^ release from the endoplasmic reticulum via IP_3_ receptors.	Maintains tissue homeostasis. Contributes to proliferation, myofibroblast differentiation, ECM synthesis, and migration to sites of injury	Ca^2+^ release enhances collagen secretion, myofibroblast differentiation, cytoskeletal polymerization, and contractility.	[259,260,261,262,263,265,268,269]
Gα12/13	AT1R, ETBR, LPA1, S1PR2, S1PR3, PAR1, PAR2, TBXA2R	Stimulates RhoA and ROCK signaling. Cytoskeletal remodeling.	Directs cell migration and wound healing. Regulates expression of genes involved in cell growth and differentiation. Assembly of actin stress fibers and focal adhesions to maintain cell shape and tension. Establish cell polarity and facilitate the formation of stable microtubules.	Inhibit LATS1/2 kinase activity through Rho-dependent actin remodeling. Enhance ROCK signaling and enhances myofibroblast differentiation Promotes ECM synthesis.	[256,259,260,261,262,263,265,268,269]
Gαs	β2-AR, A_2A_R, A_2B_R, EP2, EP4, DRD1	Activates AC which produces cAMP. cAMP activates PKA and Epac. PKA -> CREB Epac -> Rap1	Destabilize F-actin assembly within cells and promotes matrix degradation. Tissue maintenance and repair processes Reduce pro-inflammatory cytokines	Inhibition of pro-fibrotic transcription factors Increase the expression of cathepsins and Smad7 PKA increases phosphorylation of v-ATPase Increase collagen degradation Inhibition of myofibroblast differentiation	[242,257,258,270,271,272,273,274]

**Table 3:** Relative expression levels of TRPV4 in discrete tissues and implications for fibrosis.

Organ	Tissue	Relative TRPV4 RNA Expression (nTPM) *	TRPV4 Relative Protein Expression *		Implications for Fibrosis	Ref.
			Cell Type	Protein Expression (Score)		
Brain	Cerebral cortex	0.1	Endothelial cells	Not detected	TRPV4 activation triggers the release of pro-inflammatory cytokines. TRPV4 hyperactivation leads to neurotoxicity, contributing to neuronal damage associated with brain injury	[335]
			Glial cells	Low		
			Neuronal cells	High		
			Neuropil	Medium		
	Cerebellum	0.1	Cells in the granular layer	Low		
			Cells in the molecular layer	Not detected		
			Purkinje cells	Medium		
	Hippocampus	0.2	Glial cells	Low		
			Neuronal cells	High		
	Basal Ganglia	0.2	Glial cells	Low		
Endocrine tissues	Thyroid gland	0.9	Glandular cells	Medium	TRPV4 promotes the progression of pancreatic ductal adenocarcinoma by producing excessive ECM	[336]
	Parathyroid gland	0.3	Glandular cells	Low		
	Adrenal gland	0.8	Glandular cells	High		
	Pituitary gland	2.1	N/A	N/A		
Respiratory system	Nasopharynx	N/A	Respiratory epithelial cells	Low	TRPV4 mediates myofibroblast differentiation and pulmonary fibrosis. Upregulated in bleomycin-induced pulmonary fibrosis.	[337,338]
	Bronchus	N/A	Respiratory epithelial cells	Medium		
	Lung	2.2	Alveolar cells	Low		
			Macrophages	Medium		
Proximal digestive tract	Oral mucosa	N/A	Squamous epithelial cells	Not detected	TRPV4 increases Ca^2+^ influx and salivation	[339]
		N/A	Keratinocytes	Low **		
	Salivary gland	18.6	Glandular cells	Low		
	Esophagus	10.0	Squamous epithelial cells	Not detected		
	Tongue	0.0	N/A	Low **		
Gastrointestinal tract	Stomach	0.7	Glandular cells	Medium	TRPV4 activation participates in intestinal inflammation. TRPV4 antagonists show a positive protective effect. TRPV4 increases intestinal permeability	[332,340,341]
	Small intestine	0.4	Glandular cells	Medium		
	Colon	0.4	Endothelial cells	Not detected		
			Glandular cells	Low		
	Rectum	0.3	Glandular cells	Not detected		
	Duodenum	0.7	Glandular cells	High		
Liver	Liver	8.4	Cholangiocytes	Not detected	TRPV4 inhibits hepatic stellate cell apoptosis by regulating the autophagy-mediated Akt pathway activation TRPV4 Ca^2+^ influx induces mitochondrial oxidative stress	[208,342]
			Hepatocytes	Not detected		
Gallbladder	Gallbladder	1.3	Glandular cells	Not detected	TRPV4 promotes a pro-inflammatory environment	[208]
Pancreas	Pancreas	4.3	Exocrine glandular cells	Not detected	TRPV4 channel activation leads to sustained ECM deposition and differentiation into inflammatory cancer-associated fibroblasts	[343]
			Pancreatic endocrine cells	High		
Kidney and Urinary bladder	Kidney	14.3	Cells in glomeruli	Low	High lactic acid aggravates renal fibrosis and promotes TRPV4-TGFβ1-SMAD2/3 fibrotic pathway	[344]
			Cells in tubules	Low		
	Urinary bladder	1.8	Urothelial cells	Low		
Male tissues	Testis	0.7	Cells in seminiferous ducts	High	TRPV4 activation is involved in sperm motility and function. TRPV4 controls cell proliferation and ECM biosynthesis.	[345]
			Leydig cells	Medium		
	Epididymis	0.4	Glandular cells	Not detected		
	Prostate	6.6	Glandular cells	Low		
	Seminal vesicle	0.8	N/A	N/A		
Female tissues	Vagina	2.1	Squamous epithelial cells	Not detected	N/A	N/A
	Ovary	0.4	Ovarian stroma cells	Medium	Promotes proliferation and migration in ovarian cancer	[346]
	Fallopian tube	4.5	Glandular cells	Not detected	TRPV4 increases Ca^2+^ and decreases mitochondrial potential level	[347]
	Endometrium	0.6	Cells in the endometrial stroma	Medium	Substrate stiffness modifies gene expression in endometrial fibroblasts	[348,349]
			Glandular cells	Low		
	Cervix	1.9	Glandular cells	Not detected	Mechanical sensing of TRPV4 helps increase uterine contraction. TRPV4 activates inflammatory signaling	[350,351,352]
	Placenta	5.0	Decidual cells	High		
			Trophoblastic cells	Low		
	Breast	1.8	Adipocytes	Not detected	TRPV4 overexpression promoted breast cancer cell softness, blebbing, and actin reorganization	[353]
			Glandular cells	Medium		
			Myoepithelial cells	Not detected		
Muscle tissues	Heart muscle	0.8	Cardiomyocytes	Medium	TRPV4 mediates cardiac fibrosis by increasing promoter activity and fibroblast differentiation by activation of ROCK and MRTF-A pathways	[15,354]
	Smooth muscle	0.7	Smooth muscle cells	Low	Shear stress also activates TRPV4, resulting in Ca^2+^ influx that stimulates other Ca^2+^-dependent IP_3_ receptors in the endothelium and vasodilation	[208,355]
	Skeletal muscle	0.1	Myocytes	Low	Constant TRP channel expression levels promote imbalances with diminished Ca^2+^-handling in mdx muscle fibers. Enhances inflammatory response	[356]
Connective and Soft tissues	Soft tissue	N/A	Fibroblasts	Medium	Fibroblasts continuously remodel the ECM to maintain tissue health using mechanosensory properties of TRPV4. TRPV4 promotes myofibroblast differentiation and ECM synthesis	[21,61,357]
	Adipose tissue	0.9	Adipocytes	Not detected	White adipose tissue inflammation and accumulation	[358]
Skin	Skin	7.3	Epidermal cells	Not detected	TRPV4 promotes dermal myofibroblast differentiation due to matrix stiffness induction	[329,357]
			Fibroblasts	Medium		
			Keratinocytes	Low		
			Langerhans	Not detected		
			Melanocytes	Not detected		
Bone marrow and Lymphoid tissues	Appendix	0.9	Glandular cells	Not detected	TRPV4 plays a role in lymphatic inflammation and secondary lymphedema	[359]
			Lymphoid tissue	Medium		
	Spleen	2.8	Cells in red pulp	Low		
			Cells in white pulp	Not detected		
	Lymph node	0.8	Germinal center cells	Low		
			Non-germinal center cells	Low		
	Tonsil	0.9	Germinal center cells	Low		
			Non-germinal center cells	Low		
			Squamous epithelial cells	Low		
	Bone marrow	0.1	Hematopoietic cells	Not detected		

* RNA sequencing and protein expression of TRPV4 in different tissues are from ref. [360] https://www.proteinatlas.org/ENSG00000111199-TRPV4/tissue (accessed on 9 November 2025) ** Protein localization of TRPV4 in the oral mucosa and tongue is from ref. [361].

## Data Availability

This is a review paper; all the data were derived from the references provided in the text.

## Abbreviations

The following abbreviations are used in this manuscript:

AC	Adenylyl cyclase
α-SMA	Alpha-smooth muscle actin
CaMK-I	Ca^2+^/calmodulin-dependent protein kinase-II
cAMP	Cyclic adenosine monophosphate
Ca^2+^	Calcium ions
CBP	CREB-binding protein
CREB	cAMP-response element-binding protein
DAG	Diacylglycerol
DDR1	Discoidin domain receptor 1
DRD1	Dopamine receptor D1
ECM	Extracellular matrix
ERK	Extracellular signal-regulated kinase
Epac	Exchange protein activated by cAMP
FAK	Focal adhesion kinase
FliI	Flightless-I
Gαs	G protein alpha stimulatory subunit
GPCR	G protein-coupled receptor
IP_3_	Inositol-1,4,5-triphosphate
LATS1/2	Large tumor suppressor kinase ½
MAPK	Mitogen-activated protein kinase
MMP	Matrix metalloproteinase
MRTF-A	Myocardin-related transcription factor A
MT1-MMP	Membrane-type 1-matrix metalloproteinase
NMMIIA	Non-muscle myosin IIA
PDE	Phosphodiesterase
PGE_2_	Prostaglandin E_2_
PI3K	Phosphoinositide 3-kinase
PIP_2_	Phosphatidylinositol-4,5-biphosphate
ROCK	Rho-Rho-associated protein kinase
TEAD	TEA domain
TGF-β1	Transforming growth factor beta1
TIMP-2	Tissue inhibitor of metalloproteinase 2
TRP	Transient receptor potential
TRPV4	Transient receptor potential vanilloid type 4
uPARAP/Endo180	Urokinase plasminogen activator receptor-associated protein
YAP/TAZ	Yes-associated protein 1/transcriptional coactivator with PDZ-binding motif
